# HNCDB: An Integrated Gene and Drug Database for Head and Neck Cancer

**DOI:** 10.3389/fonc.2019.00371

**Published:** 2019-05-14

**Authors:** Qingbin Zhang, Xingyang Li, Xuan Su, Hongwan Zhang, Hanbing Wang, Sanjun Yin, Xiaoqing Pei, Ankui Yang, Zhixiang Zuo

**Affiliations:** ^1^Key Laboratory of Oral Medicine, Guangzhou Institute of Oral Disease, Stomatology Hospital of Guangzhou Medical University, Guangzhou, China; ^2^State Key Laboratory of Oncology in South China, Sun Yat-sen University Cancer Center, Collaborative Innovation Center for Cancer Medicine, Sun Yat-sen University, Guangzhou, China; ^3^Department of Cancer Biology, Health Time Gene Institute, Shenzhen, China

**Keywords:** head and neck cancer, gene, drug, connectivity map, expression analysis

## Abstract

Head and neck cancer (HNC) is the sixth most common cancer worldwide. Over the last decade, an enormous amount of well-annotated gene and drug data has accumulated for HNC. However, a comprehensive repository is not yet available. Here, we constructed the Head and Neck Cancer Database (HNCDB: http://hncdb.cancerbio.info) using text mining followed by manual curation of the literature to collect reliable information on the HNC-related genes and drugs. The high-throughput gene expression data for HNC were also integrated into HNCDB. HNCDB includes the following three separate but closely related components: “HNC GENE,” “Connectivity Map,” and “ANALYSIS.” The “HNC GENE” component contains comprehensive information for the 1,173 HNC-related genes manually curated from 2,564 publications. The “Connectivity Map” includes information on the potential connections between the 176 drugs manually curated from 2,032 publications and the 1,173 HNC-related genes. The “ANALYSIS” component allows users to conduct correlation, differential expression, and survival analyses in the 2,403 samples from 78 HNC gene expression datasets. Taken together, we believe that HNCDB will be of significant benefit for the HNC community and promote further advances for precision medicine research on HNC.

## Introduction

Head and neck cancer (HNC) is characterized by tumors that occur in the head and neck regions, excluding the eyes, brain, ears, thyroid, and esophagus. In the past 10 years, the incidence of head and neck cancer has increased significantly, with an average of ~600,000 new patients per year, and has become the sixth most common cancer in the world today (1). Based on its underlying cause, head and neck cancer is divided into the following two major categories: HPV-negative head and neck cancer associated with smoking and drinking and HPV-positive head and neck cancer caused by high-risk HPV. According to the size and developmental stage of the head and neck cancer tumor, the main treatment methods are surgery, chemotherapy, radiotherapy, molecular targeted therapy and multimethod combination therapy (2). Although concurrent chemoradiotherapy can greatly promote effective treatment of head and neck cancer, a considerable number of patients are intolerant to this treatment. Cetuximab is a targeted drug for EGFR and is the only molecularly targeted therapy for head and neck cancer, but cetuximab is only effective in 10–13% of patients with head and neck cancer (3). At present, the 5-year survival rate of patients with head and neck cancer is still only 50–60%. Therefore, new treatment methods are needed to enrich the treatment of head and neck cancer and improve the survival rate of patients with head and neck cancer, which necessitates the understanding of the underlying molecular biology of HNC.

Over the last decade, research in HNC has led to an enormous amount of well-annotated data on the molecular biology of HNC that is freely accessible at PubMed, which is maintained by the US National Library of Medicine. However, PubMed is not specifically designed for HNC, and it is tedious and time-consuming to find the information of interest. Several HNC-specific databases have been established to solve this problem. HNOCDB is a comprehensive database of genes relevant to HNC and was constructed based on text mining on PubMed (4). HNdb is an integrated database of gene and protein information that covers genomics, transcriptomics, proteomics, and literature evidence for HNC (5). OrCGDB is a database of genes involved in oral cancer (6). However, none of these databases provide insights into the treatment of HNC. To successfully treat a disease, the disease symptoms ultimately need to be connected with altered gene function(s) and clinically used drugs, thus highlighting the necessity of understanding disease-specific gene-drug relationships.

The rapid development of high-throughput technology methods, such as RNA sequencing and microarray, has resulted in large amounts of transcriptomic data on HNC. The gene expression analysis of transcriptomic data is useful for understanding cancer biology and finding candidate drug targets. The NCBI GEO database and the Cancer Genome Atlas (TCGA) projects host transcriptomic data for tens of thousands of cancer samples. However, there is still a gap between cancer genomic data and data mining for users without high-throughput analysis skills. In particular, a database that integrates knowledge from literature text mining and high-throughput data is lacking for HNC.

**Figure 1:**
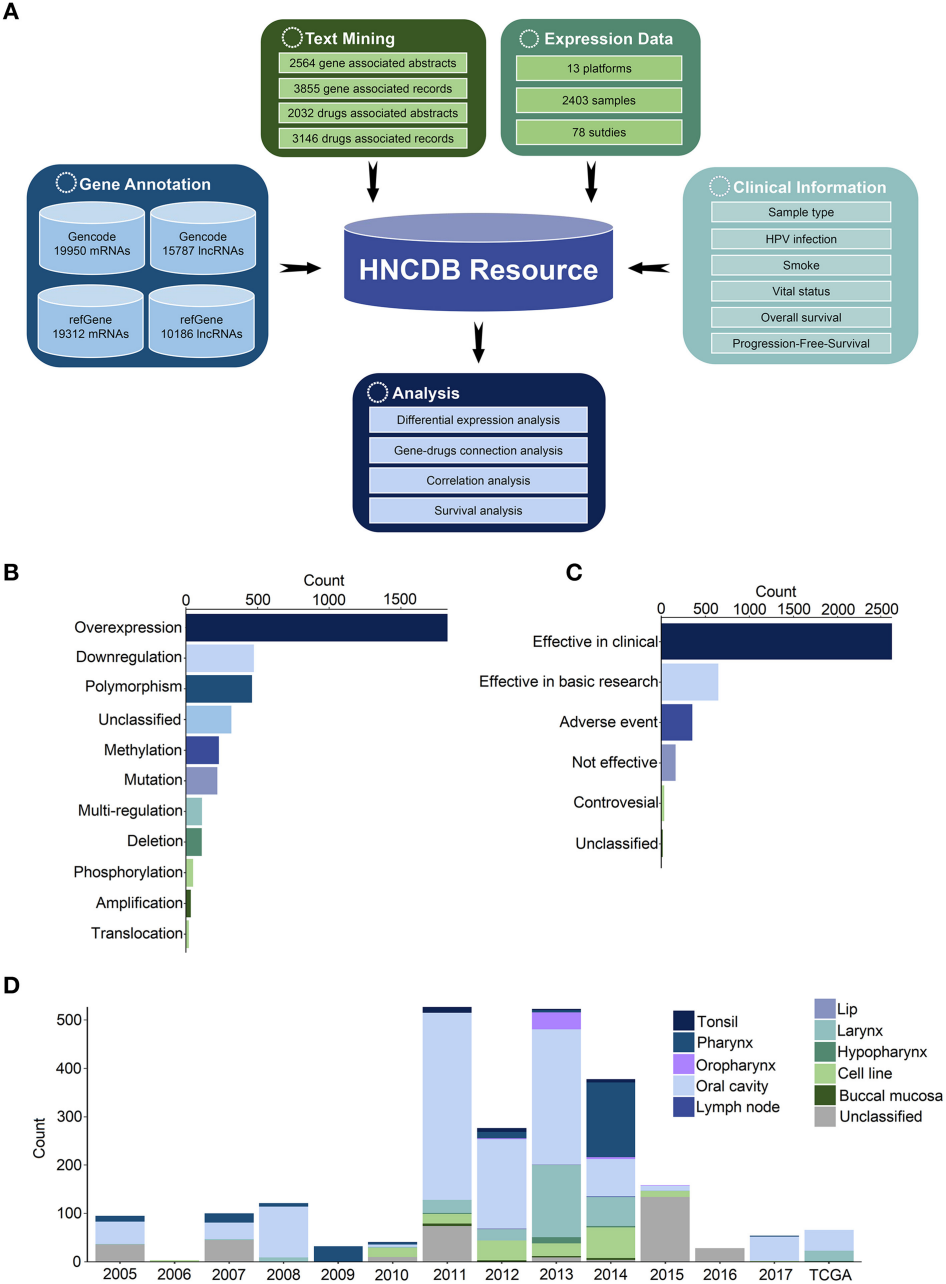
Construction of HNCDB. **(A)** Flowchart of the database construction process. **(B)** HNC-related abstracts associated with gene dysregulation in HNC. **(C)** HNC-related abstracts associated with drug efficacy in HNC. **(D)** Summary of the HNC gene expression dataset in HNCDB.

Here, we present an integrated database called the Head and Neck Cancer Database (HNCDB) dedicated to addressing the above issues. We constructed HNCDB by integrating literature text mining of PubMed abstracts and high-throughput gene expression data collected from the GEO and TCGA databases for HNC (Figure 1A). The literature text mining process was followed by manual curation to collect reliable information on HNC-related genes and drugs. R packages were used to implement the gene expression analysis methods. The database is available at http://hncdb.cancerbio.info.

## Materials and Methods

### Collection of HNC-related Genes and Drugs

HNC-related genes and drugs were collected using the previous reported strategy (7). Briefly, HNC-related abstracts were extracted from NCBI PubMed using the disease-related keywords for HNC, such as “HNC,” “HNCSCC,” “head and neck cancer,” “oral cancer,” “throat cancer,” “laryngeal cancer,” “pharyngeal cancer,” and “oropharyngeal cancer.” Each abstract was scanned for genes and drugs obtained from HGNC (8), ENTREZ Gene (9), OMIM (10), UniProt (11), and DrugBank (12). As a result, 48,324 abstracts were found to contain gene information, and 40,478 abstracts were found to contain drug information. Then, a text mining strategy was then applied to assess the gene-disease association and drug-disease association in these abstracts. We calculated the score of the gene-disease associations (SGDs) and drug-disease associations (SDDs) as follows:

(1) $$\begin{array}{l} SGD=N_{c}\times w_{c}+FLAG_{gene}\times N_{hnc}\times w_{hnc}+FLAG_{hnc}\\ \;\times N_{gene}\times w_{gene} \end{array}$$

(2) $$\begin{array}{l} SDD=N_{c}\times w_{c}+FLAG_{drug}\times N_{hnc}\times w_{hnc}+FLAG_{hnc}\\ \;\times N_{drug}\times w_{drug} \end{array}$$

where *N*_*c*_ is the number of sentences containing both HNC-relevant and gene/drug-relevant terms, and *w*_*c*_ is the corresponding weight; *N*_*hnc*_ is the number of sentences containing only HNC-relevant terms, and *w*_*hnc*_ is the corresponding weight; *N*_*gene*_ is the number of sentences containing only gene-relevant terms, and *w*_*gene*_ is the corresponding weight; and *N*_*drug*_ is the number of sentences containing only drug-relevant terms, and *w*_*drug*_ is the corresponding weight. *FLAG*_*gene*_ indicates if any gene-relevant terms exist in the whole abstract. *FLAG*_*gene*_ = 1 means that gene-relevant terms exist, while *FLAG*_*gene*_ = 0 means that gene-relevant terms do not exist. *FLAG*_*drug*_ indicates if any drug-relevant terms exist in the whole abstract. *FLAG*_*drug*_ = 1 means that drug-relevant terms exist, while *FLAG*_*drug*_ = 0 means that drug-relevant terms do not exist.

We developed a web server to facilitate the curation process. For gene curation in each abstract, the keywords indicating disease name, gene name, molecular event, and molecular function were highlighted, and the candidate genes were listed for manual review (Figure S1). For drug curation in each abstract, the keywords indicating disease name, drug name, and drug efficacy were highlighted, and the candidate drugs were listed for manual review (Figure S2). The corresponding evidence of molecular and genetic changes from each abstract was also collected. A MySQL relational database system was built to store the curated information.

### Collection of HNC-related Expression Datasets

The HNC-related keywords were used to query the NCBI GEO database. The microarray expression datasets together with the clinical data were downloaded. All the data were manually curated. Additionally, we collected processed expression data of the TCGA samples from Broad Institute's Firehose (13) and TANRIC (14) databases.

### Probe Reannotation

All probe sequences from the different platforms were downloaded from the official website. The probes were reannotated following the previously reported procedures (15). First, all the probe sequences from the different platforms were aligned to the human genome (GRCh38) using a BLAST-like alignment tool (BLAT) (16). Only the alignments with no more than one mismatch, no gaps and a similarity score larger than 90 were preserved. In general, probes (50–60 nucleotides) from Agilent, Illumina, and other companies were designed to locate target genes or transcripts. The microarrays from Affymetrix utilized a probe set containing a group of 25-mer probes to represent a gene or transcript. Thus, for the Affymetrix data, we combined probes that specifically corresponded to the same probe set and ensured that there were at least 3 perfectly matching and adjacent probes in each probe set. Second, we mapped the probes to their coding genes or lncRNAs according to their genomic coordinates. If any probes were targeted to both coding genes and lncRNAs, we only preserved the annotation of the coding genes.

### Normalization of the Microarray Expression Data

For the Affymetrix data, raw CEL files were obtained when possible and normalized with the RMA algorithm (“affy” package v. 1.26 (17) or “apt-probeset-summarize” in Affymetrix Power Tools v. 1.19.0). The datasets from Agilent, Illumina, and other platforms were quantile-normalized with the Limma package (18). For certain studies that lacked raw data, we used the data matrix provided in the GEO database as a normalized expression matrix. In addition, each gene was log2 transformed if it was not already in log space. Next, all the probes were transformed to gene names based on the reannotation file. The average expression was calculated to represent this gene when multiple probes targeted a given gene.

### Differential Expression Analysis

Tumor samples vs. normal samples, HPV-positive samples vs. HPV-negative samples and smokers vs. non-smokers were used as the categories for differential expression analysis. For each condition, the differential expression analysis was performed with the Limma package (18). *P* < 0.05 was used as the significance threshold. We utilized a robust rank aggregation algorithm to integrate the differential expression analysis results from different microarray studies in an unbiased manner (19). The aggregation rank score (AR score) represents the integrated rank from the meta-analysis of the fold-change in the different microarray studies.

### Building the HNC-related Drug-Gene Connectivity Map

The HNC-related genes and drugs collected from text mining were used to build a drug-gene connectivity map modified from a previous study (20). Briefly, we assigned a connectivity score, θ_*gd*_, for each possible pair of HNC-related genes and drugs using a regularized log-odds function as shown in formula (3):

(3) $$\begin{array}{r} \theta gd=\text{ln}(\frac{df_{gd}\times P+\lambda }{d_{fg}\times df_{d}+\lambda })\times (1+Corr_{gd}) \end{array}$$

where *df*_*gd*_ is the number of PubMed abstracts in which gene and drug terms co-occurred; *df*_*g*_ and *df*_*d*_ are the number of PubMed abstracts in which gene g and drug d are mentioned, respectively; *P* is the number of total PubMed abstracts related to HNC; *Corr*_*gd*_ is the average absolute Pearson correlation between the expression of drug d targets and gene g in all the gene expression datasets; and λ is a small constant introduced to avoid domain errors—and here, we let λ = 1.

### Survival Analysis

The R package “survival” was used to perform survival analysis. Univariate Cox proportional hazards regression analysis was performed to assess the associations between survival and gene expression in the datasets we collected containing overall survival data or progression-free survival data.

### Implementation of a Web Server

A Linux server hosted the MySQL database, and JavaScript was used to build the web page.

## Results

### Data Summary

There were 1,173 genes implicated in dysregulation (such as overexpression, downregulation, polymorphism, DNA methylation, mutation, deletion, amplification and translocation) in HNC from 2,564 abstracts. In total, 176 drugs associated with dysregulation were shown to be effective as HNC treatment (such as effective in clinical trials, effective in basic research, and no adverse events and controversial effects) from 2,032 abstracts (Figures 1B,C). In addition, 78 expression datasets in 13 platforms containing 2,403 samples were obtained from the NCBI GEO and TCGA databases (Figure 1D). The probes were reannotated to map their protein-coding genes or lncRNAs. In total, 17,883 coding genes and 4,158 lncRNAs were identified in all samples.

### Web Interface and Usage

HNCDB is available at http://hncdb.cancerbio.info and includes the following three separate but closely related components: “HNC GENE,” “Connectivity Map,” and “ANALYSIS.”

#### HNC Gene

**Figure 2:**
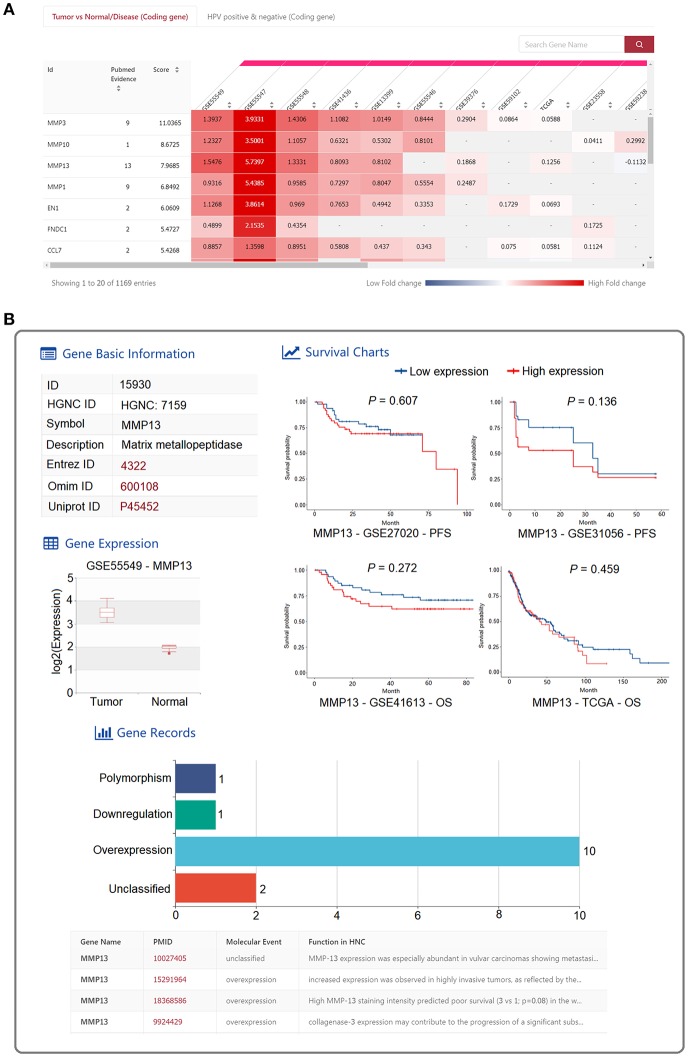
Web interface for “HNC GENE” in HNCDB. **(A)** An interactive heatmap displaying the differential expression results of the 1,173 HNC-related genes across all the HNC gene expression datasets. **(B)** Detailed information of a selected gene in a certain dataset.

An interactive heatmap was implemented to present the differential expression results of the 1,173 HNC-related genes across all the datasets (Figure 2A). Differential expression analyses were performed between tumor and normal HNC samples or between HPV-positive and HPV-negative HNC samples. The log2 (fold change) value from the differential expression analysis of each dataset was presented in the interactive heatmap. A meta-score obtained by aggregating the fold change from all the datasets using R package “RobustRankAggreg” (19) was provided in the heatmap. Moreover, the number of PubMed abstracts supporting each HNC-related gene was also provided. Users are able to browse the differentially expressed HNC-related gene in each dataset with search and sort functions. A search box helps users quickly locate HNC-related genes of interest. Furthermore, users can sort the HNC-related genes based on the meta-scores, number of supporting PubMed abstracts and fold change values derived from each study. The detailed information for the differential expression of the HNC-related gene of interest in a certain dataset was shown when the box representing the fold change is clicked (Figure 2B). The detailed information is composed of the following four sections: “Gene Basic Information” contains the gene name and external database IDs of the selected gene; “Gene Records” contains the PubMed abstracts that contain the detailed information about the relationship between the selected gene and HNC; “Gene expression” contains a boxplot showing the differential expression between tumor and normal samples or between HPV-positive and HPV-negative HNC samples; and “Survival” shows the Kaplan-Meier survival plots describing the association between the expression of the selected gene and survival.

#### Connectivity Map

**Figure 3:**
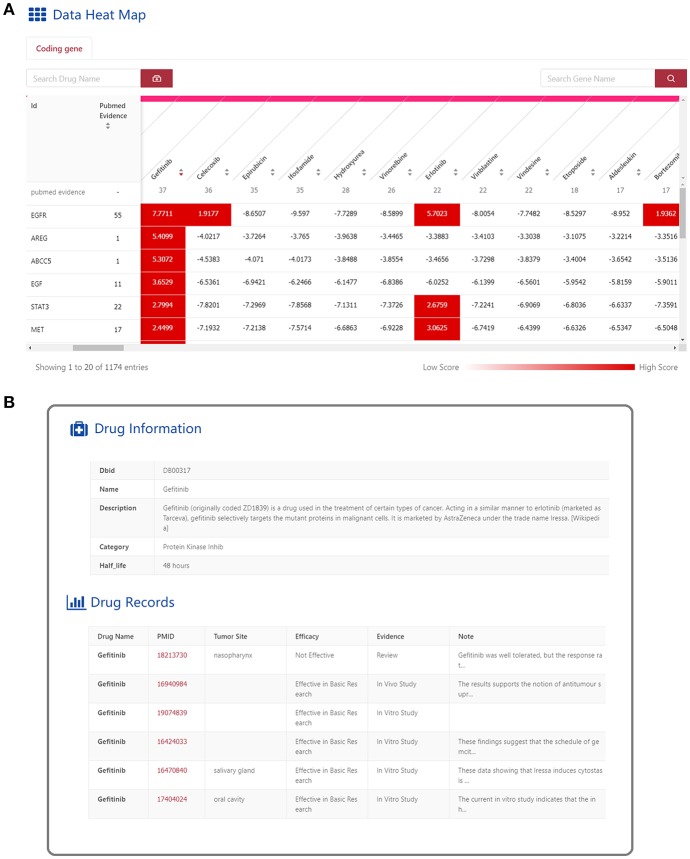
Web interface for “Connectivity Map” in HNCDB. **(A)** The interactive heatmap of the “connectivity score” for the HNC-related gene and drug pair. **(B)** Detailed information of a selected HNC-related gene-drug pair.

The “Connectivity Map” is designed to connect the HNC-related genes and drugs in the context of HNC disease, which is expected to provide insights into potential new strategies for HNC treatment. An interactive heatmap was implemented to present the Connectivity Map (Figure 3A). The 1,173 HNC-related genes are shown as rows of the heatmap, and the 176 HNC-related drugs are shown as columns. Each cell in the heatmap is the “connectivity score” that represents the association strength of the gene-drug pair. In addition, the number of PubMed abstracts that contain the HNC-related gene and drug are displayed in the first column and the first row, respectively. By clicking the number, users able to check the details of papers that discuss the genes or drugs of interest. Users can explore the “Connectivity Map” by searching for the genes or drugs of interest in the search box. By clicking the “connectivity score” cell, users can obtain detailed information about the connection of the selected genes and drugs (Figure 3B). The detailed information for the connectivity map includes six sections. “Drug Information” shows the basic information from DrugBank about the selected drug. “Drug Records” shows the PubMed evidence supporting the association between the selected drug and HNC. Moreover, gene-related information, such as “Basic Gene Information,” “Gene Records,” “Gene Expression,” and “Survival” are also shown for the selected gene as described above.

#### Analysis

**Figure 4:**
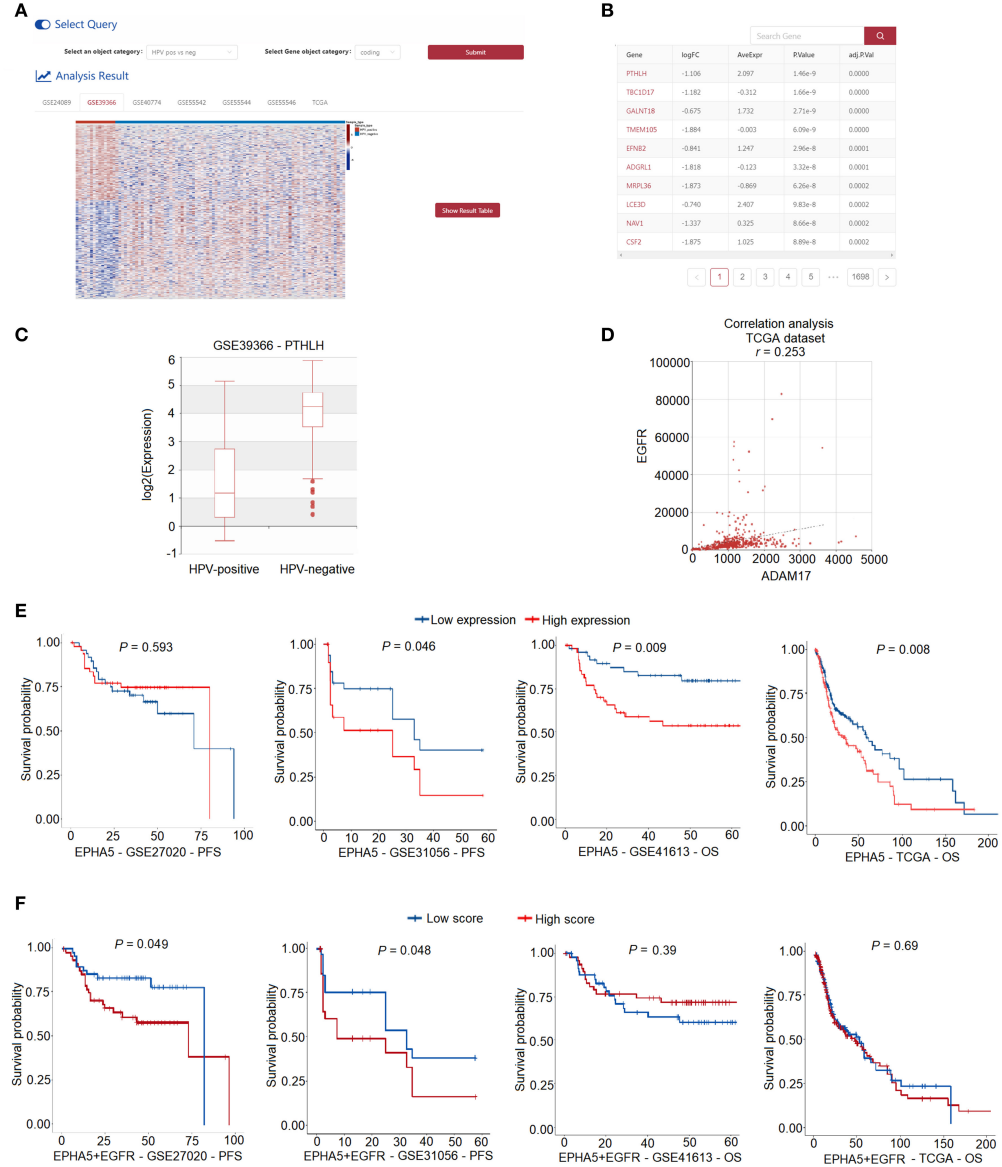
Web interface for “ANALYSIS” in HNCDB. **(A)** A heatmap showing all the differentially expressed genes in one selected HNC gene expression dataset. **(B)** Detailed information of the results of the differential expression analysis. **(C)** Box plot showing the differentially expressed gene of interest in the selected dataset. **(D)** Scatter plot showing the result of the correlation analysis. **(E)** KM plot showing the result of the univariate survival analysis. **(F)** KM plot showing the result of the multivariate survival analysis.

The “ANALYSIS” module implements three analysis methods including “differential expression analysis,” “correlation analysis,” and “survival analysis.” Users can interactively perform these analyses on 2,403 HNC samples collected from the GEO and TCGA databases. “Differential expression analysis” allows users to perform differential expression analysis between “HPV-positive” and “HPV-negative” samples or “tumor” and “normal” HNC samples. A heatmap shows the significant differentially expressed genes in the selected dataset (Figure 4A). Users can click “Show Result Table” to browse more details of the differential expression analysis results (Figure 4B). A boxplot representing the differential expression of a gene of interest is displayed when users click on the gene name in the table (Figure 4C). “Correlation analysis” allows users to investigate the coexpression between two genes in the selected dataset. The correlation between the two genes is shown as a scatter plot (Figure 4D). In “survival analysis,” users can obtain the results of a univariate Cox proportional hazards regression analysis to assess the relationship between survival and the expression of the gene of interest (Figure 4E). In addition, users can input multiple genes of interest to perform multivariate Cox model survival analysis (Figure 4F).

### An Application Example: Oncogenic Role of *EGFR* in Head and Neck Cancer and its Clinical Implication, as Revealed by HNCDB

**Figure 5:**
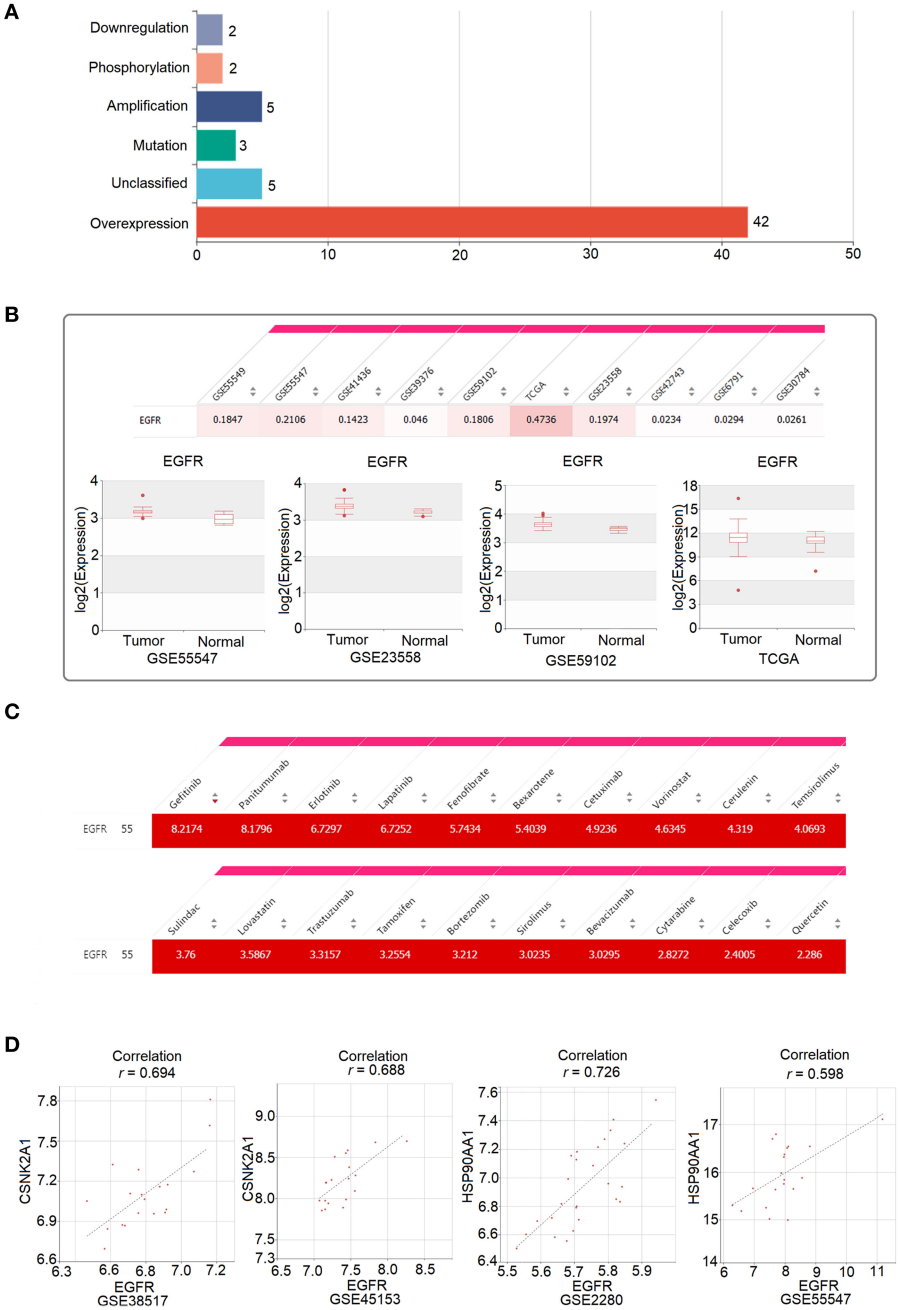
Oncogenic role of *EGFR* in head and neck cancer and its clinical implication, as revealed by HNCDB. **(A)** The PubMed abstracts indicating a relationship between EGFR and HNC. **(B)** Significant upregulation of EGFR in tumor samples compared to in normal samples in the HNC gene expression datasets collected in HNCDB. **(C)** The connectivity score for the identified drugs and EGFR. **(D)** Correlation between EGFR and the two targets of quercetin (CSNK2A1 and HSP90AA1).

*EGFR*, known as epidermal growth factor receptor, is a member of the erbB family of receptor tyrosine kinases. EGFR is highly expressed in HNC, and its expression is associated with poor outcomes in patients with HNC (21, 22). The overexpression of *EGFR* activates signaling pathways that lead to cell division, the inhibition of apoptosis, cell invasion, and angiogenesis (23). Consistent with previous studies, 55 HNC-related PubMed abstracts indicated that *EGFR* was dysregulated in HNC, and 42 out of the 55 PubMed abstracts showed that *EGFR* was overexpressed in HNC (Figure 5A). Moreover, *EGFR* was found to be significantly upregulated in tumor samples compared to normal samples, as shown in 10 of the 23 HNC datasets collected in HNCDB (Figure 5B).

EGFR is the most validated molecular target in the treatment of HNC. The monoclonal antibody cetuximab is an approved drug that targets EGFR in HNC and is associated with increased survival after chemotherapy for metastatic or recurrent disease and after radiation therapy for locally advanced disease (24, 25). Consistently, as shown in the “Connectivity Map” of HNCDB, cetuximab and EGFR have high connectivity scores (Figure 5C). Among all the drugs that have high connections with EGFR, the drugs cetuximab, gefitinib, erlotinib, panitumumab, lapatinib, and trastuzumab directly target EGFR as recorded in the DrugBank database (Figure 5C). In addition, some drugs that have not been shown to directly target EGFR, such as quercetin, also have a high connection with EGFR (Figure 5C). Quercetin is a flavonoid natural product found in many foods and is effective for metastatic inhibition in HNC (26). We hypothesized that quercetin might be effective in HNC through the indirect targeting of EGFR. Indeed, we observed that EGFR was highly positively correlated with the expression of HSP90AA1 and CSNK2A1, which are two direct targets of quercetin that were identified by LC MS/MS (27) (Figure 5D).

## Discussion

First, HNCDB is an integrated knowledgebase for HNC-related genes. By using HNCDB, users can obtain an overview of the knowledge about HNC simply, quickly and accurately. The HNC-related genes along with evidence from PubMed are presented together with gene expression data in an interactive heatmap. Through some mouse clicking, users can quickly find information in the literature about the genes of interest and the gene expression pattern in HNC samples.

The ultimate goal in biomedical research is to establish networks that connect genes and drugs in the context of a specific disease, thereby providing effective drug candidates to treat the disease. To this end, in 2006, Lamb et al. proposed the concept of a “Connectivity Map” that connects the small molecules and gene patterns associated with a specific disease (28). Since then, various connectivity map projects have been undertaken to provide systemic solutions for the discovery of functional connections between drugs, genes and diseases, as well as drug discovery and development (29, 30). However, this approach is time-consuming and costly since it requires the high-throughput systematic screening of small molecules in human cell lines. We endorsed a computational strategy to build the connectivity map for HNC based on the highly curated knowledge in the literature about HNC-related genes and drugs (20). The connectivity map we implemented in HNCDB confirmed the known gene-drug relationship in HNC. Moreover, it also suggested new potential targeted therapy strategies for further experimental validation. For instance, we found that EGFR had the highest connectivity score in the connectivity map. This is consistent with the fact that epidermal growth factor receptor (EGFR) inhibitors are the only targeted agents that have been approved to treat HNC so far. Moreover, we found that EGFR showed a high connectivity score with 20 drugs, among which gefitinib, panitumumab, erlotinib, lapatinib, cetuximab, and trastuzumab are known drugs that target EGFR, as recorded in DrugBank. Some drugs, such as quercetin, have no evidence to support that they directly target EGFR but show a high connection. Quercetin is a major dietary flavonol that exists in many fruits and vegetables. Previous studies have shown that quercetin has potential therapeutic effects in cancers including HNC, prostate cancer and breast cancer. Quercetin has been shown to inhibit cancer cell growth through targeting EGFR in HNC cancer cells (31). Taken together, the “Connectivity Map” in HNCDB could serve as a useful tool to provide potential hypotheses for treating HNC in both basic research and clinical studies.

Another important application of HNCDB is that it can enable HNC researchers who lack high-throughput analysis experience to explore high-throughput transcriptomic data in a convenient manner. HNCDB implements many gene expression analysis methods in the “ANALYSIS” section. By using the “ANALYSIS” section, users can easily test their hypotheses through differential expression analysis, correlation analysis and survival analysis. Moreover, users can discover new ideas by exploring HNCDB.

In conclusion, HNCDB is a useful resource for the HNC research community. HNCDB provides a comprehensive collection of well-curated HNC-related genes and drugs as well as their connectivity maps and interactive interfaces for gene expression analysis, all of which enables researchers to better understand the cancer biology of HNC and develop useful clinical treatments for patients with HNC. In the future, HNCDB will be continuously updated. New HNC-related genes and drugs, gene expression data, and other high-throughput data, such as whole-genome/exome sequencing and methylation data, will be added to HNCDB. We believe that HNCDB has the potential to become a routinely used knowledgebase for HNC research.

## Data Availability

Publicly available datasets were analyzed in this study. This data can be found here: http://hncdb.cancerbio.info.

## Author Contributions

ZZ, AY, and XP: study concepts. ZZ: study design. QZ, XL, XS, and HZ: data acquisition. QZ, XL, and HW: quality control of data and algorithms. XL and ZZ: data analysis and interpretation. XL: statistical analysis. ZZ and XL: manuscript preparation. All authors have reviewed and edited the manuscript.

### Conflict of Interest Statement

The authors declare that the research was conducted in the absence of any commercial or financial relationships that could be construed as a potential conflict of interest.

## Supplementary Material

Figure S1 HNCDB gene curation. The keywords indicating tumor site, gene name and molecular event are highlighted with different colors. A curation box shows the drug-disease association information extracted by the curator.

Figure S2 HNCDB drug curation. The keywords indicating tumor site, drug name and drug efficacy are highlighted with different colors. A curation box shows the drug-disease association information extracted by the curator.
