# Supplementary figures and images for: HNCDB: An Integrated Gene and Drug Database for Head and Neck Cancer
Source: Front Oncol. 2019 May 14;9:371. doi: 10.3389/fonc.2019.00371 (PMC6527845; doi:10.3389/fonc.2019.00371)

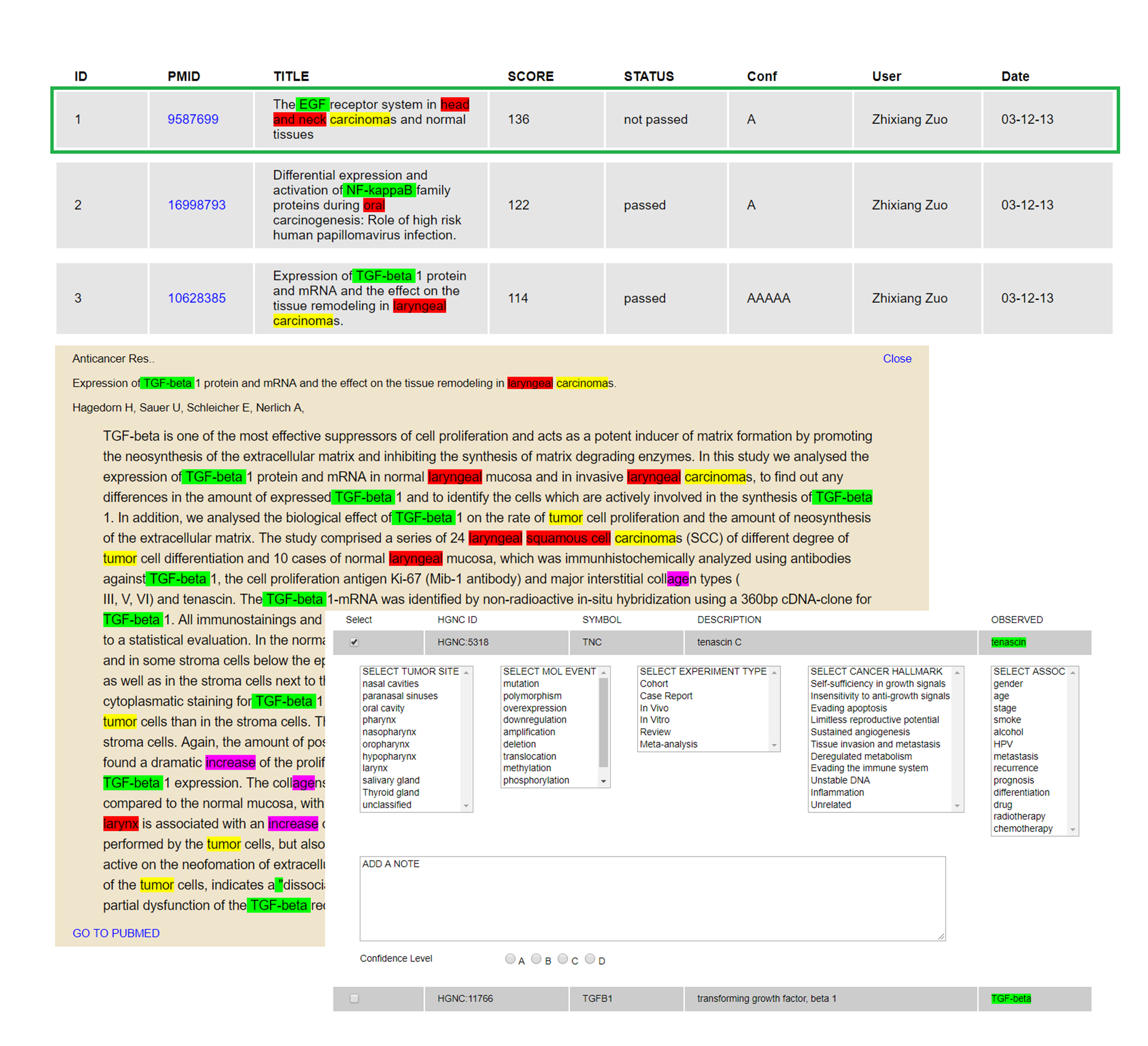

Supplement: Figure S1 — HNCDB gene curation. The keywords indicating tumor site, gene name and molecular event are highlighted with different colors. A curation box shows the drug-disease association information extracted by the curator. [file Image_1.TIF]

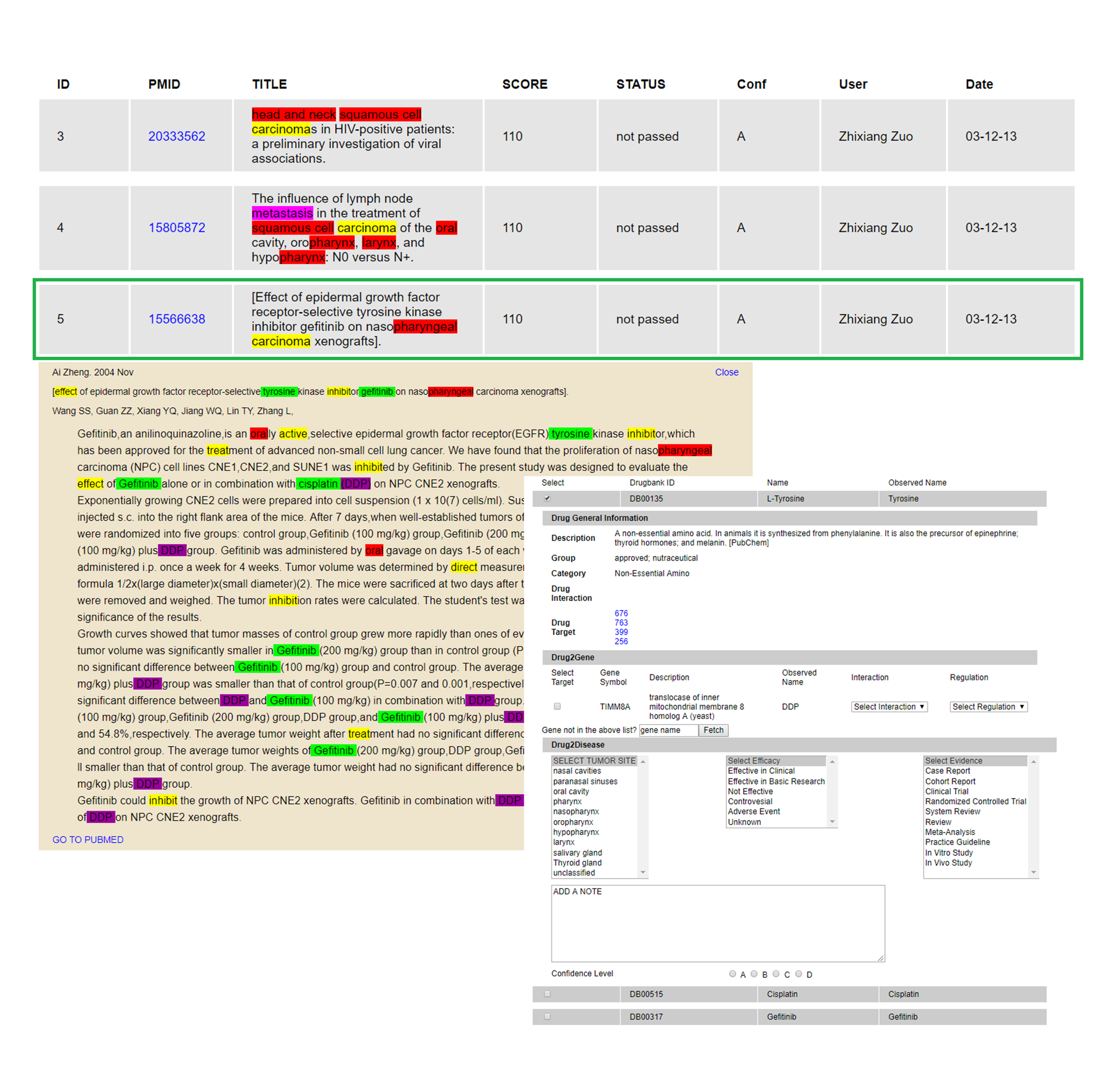

Supplement: Figure S2 — HNCDB drug curation. The keywords indicating tumor site, drug name and drug efficacy are highlighted with different colors. A curation box shows the drug-disease association information extracted by the curator. [file Image_2.TIF]
